# Study on Microstructure Evolution and Deformation Failure Mechanism of PTFE-Cu Composites Under Compression Load

**DOI:** 10.3390/polym17101380

**Published:** 2025-05-17

**Authors:** Siman Guan, Zhijun Wang, Xuezhi Tang, Ruijie Hao, Jianya Yi

**Affiliations:** 1 School of Mechatronic Engineering, North University of China, Taiyuan 030051, China; 2 Chongqing Hongyu Precision Industry Group Co., Ltd., Chongqing 402760, China

**Keywords:** PTFE-Cu, crystallinity, quasi-static compression, molecular dynamics simulation, microstructure evolution mechanism

## Abstract

In order to study the microstructure evolution of polytetrafluoroethylene–copper (PTFE-Cu) composites under compression load and reveal the molecular dynamics mechanism of deformation failure, three PTFE-Cu composites with different densities (3.0 g/cm^3^, 3.5 g/cm^3^, 4.0 g/cm^3^) were prepared in this study. The crystallinity of PTFE in each sample was determined via differential scanning calorimetry (DSC). The quasi-static compression mechanical properties test was carried out to analyze the effect of PTFE crystallinity on the macroscopic mechanical response of the composites. It is found that the crystallinity of the three PTFE-Cu composites was 43.05%, 39.49% and 40.13%, respectively, showing a non-monotonic trend of decreasing first and then increasing with an increase in copper powder content. The elastic modulus and yield strength of the material are negatively correlated with the crystallinity. The failure mode is the axial splitting failure and the composite morphology of axial splitting failure and shear tearing. Finally, the molecular dynamics simulation method is used to reveal the microstructure evolution and deformation failure mechanism of PTFE-Cu composites under compression load from the atomic scale, which provides a theoretical basis and experimental support for understanding the mechanical properties of PTFE-Cu composites.

## 1. Introduction

Polytetrafluoroethylene (PTFE), as a representative semi-crystalline polymer material, exhibits excellent physical and chemical properties due to its unique molecular structure, including excellent ductility, high chemical stability, excellent resistance to degradation and extremely low friction coefficient. These characteristics make it show broad application potential in high-tech fields, such as chemical materials [1,2], biomedical [3,4], electrical insulation [5,6], aerospace lightweight components [7,8,9] and key components in national defense equipment [10,11,12,13]. With the continuous improvements in material performance requirements in modern industry, scholars have carried out systematic research on the mechanical response mechanism of PTFE and its composite systems in recent years, especially focusing on key scientific issues such as mechanical behavior, interface effect and failure mode under load. These studies not only provide a theoretical basis for the composition control of PTFE and its composites but also lay a scientific foundation for promoting its engineering application under extreme conditions.

In the field of PTFE material performance research, mechanical performance characterization has always been the core research direction. Eric N. Brow et al. [14,15] carried out tensile and fracture tests of PTFE materials, discussed the fracture behavior of PTFE and the influence of crystallinity on fracture, and used a single sample normalization technique to provide the J-integral fracture toughness of PTFE at a certain temperature and loading rate. It was found that the crack propagation of PTFE has a strong correlation with the brittle to ductile crack propagation behavior related to two room-temperature phase transitions. Rae P et al. [16,17] studied the basic characteristics of PTFE materials and the tensile and compressive responses at different temperatures and strain rates, and they explained the causes of related mechanical responses from the microscopic scale and the failure mechanisms under different phases and phase transitions. Philip J. Rae et al. [18] studied the role of pressure-induced phase transition in the dynamic failure of Taylor cylindrical PTFE samples. By changing the temperature of the sample during the experiment, it was found that the failure of the sample may be related to the phase transition. As the temperature decreases, the ductile–brittle transition rate increases, and the fracture toughness of the material decreases. The above research not only deepens the understanding of the mechanical properties of PTFE materials but also provides important theoretical support for the engineering application of such materials under extreme working conditions.

Existing studies have shown that single polytetrafluoroethylene (PTFE) material has limitations in mechanical properties. In view of this, many scholars are committed to optimizing its mechanical properties by adding metal particles to the PTFE matrix. Among them, active composite materials based on PTFE/Al [19,20,21] have become a hot research field. Chao Ge [22,23] found that the compressive stress–strain curve of aluminum particle-filled polytetrafluoroethylene (PTFE/Al) active material showed typical elastic–plastic behavior. And tungsten powder was added to the PTFE/Al active material. Three kinds of polytetrafluoroethylene/aluminum/tungsten reactive materials with different component mass ratios were studied. It was found that the three materials failed under very low strain during quasi-static and dynamic compression. In order to study the effect of oxidants on the mechanical response and reaction characteristics of PTFE/Al active materials under dynamic impact, Jiaxiang Wu et al. [24] prepared PTFE/Al/Fe_2_O_3_, PTFE/Al/MnO_2_ and PTFE/Al/MoO_3_ reactive material samples. Through the split Hopkinson pressure bar test, it was found that the three oxidants had significant effects on the mechanical behavior and reaction characteristics of the reactive materials. Among them, PTFE/Al/Fe_2_O_3_ had the highest strength and PTFE/Al/MoO_3_ had the lowest strength. Although composition is one of the key factors affecting the mechanical properties of the material, the influence of the preparation process on the mechanical properties of the material cannot be ignored. Feng Yue Xu et al. [25] carried out quasi-static compression experiments of PTFE/Al/W reactive material samples under two preparation methods of cold isostatic pressing without sintering and pressing before sintering. It was found that the sintered samples showed higher failure stress and greater fracture toughness than the pressed samples. For the purpose of further exploring the influencing factors and failure mechanism of mechanical properties of materials, the concept of thermodynamics is introduced to explain the mechanical response of materials. Huanguo Guo et al. [26] studied the PTFE/Al/Cu/Pb (14 wt%/9 wt%/57 wt%/20 wt%) reactive material mixture using a differential scanning calorimeter (DSC), thermogravimetric analyzer (TG) and X-ray diffractometer (XRD), revealing that the actual reaction heat of the composite material not only includes the oxidation reaction between Al and PTFE decomposition product C_2_F_4_ but also includes the intermetallic compound reaction between excess Al and Cu powder. Haifu Wang et al. [27] focused on the effects of sintering and cooling processes on the geometric distortion and mechanical properties of PTFE/Al materials and found a mechanical response characteristic transition from brittleness to ductility. Combined with the thermodynamic properties of polytetrafluoroethylene and the theory of unsteady heat conduction, they clarified the internal mechanism of cooling-induced morphology change, temperature-induced distortion and strength reduction.

In addition to the active material system, many scholars have also carried out research on the mechanical response characteristics of PTFE/Cu inert composites. Early PTFE-Cu materials were used in friction units due to their low friction coefficient and high wear resistance [28,29,30]. Samuel Beckford et al. [30] found that the addition of Cu nanoparticles to PTFE film can further improve its wear resistance. Xie Ting et al. [31] discussed the influence of the shape, content and size of copper powder on the tribological properties of PTFE composites through experimental research. It is considered that both irregular copper powder and spherical copper powder can effectively reduce the wear of the composites. In addition, PTFE-Cu is also applied to the liner design of shaped charge warheads due to its inert characteristics [11,32,33,34]. The damage effect of a shaped charge warhead mainly depends on the mechanical properties of PTFE-Cu composites. The static and dynamic mechanical properties of the composites can be effectively regulated by doping a specific mass fraction of copper powder into the low-density PTFE matrix [35]. Xuezhi Tang et al. [36,37] prepared PTFE/Cu composite samples via various processes and explored the influence mechanism of preparation methods on the compressive mechanical properties of composites. The results show that sintering can effectively improve the bonding strength of PTFE and the Cu interface, and the influence of the preparation process on mechanical properties is more direct and significant than that of density. These studies provide an important theoretical basis for optimizing the structure–property relationship of PTFE/Cu inert composites.

With the development of computer science and technology, molecular dynamics (MD) simulation technology has the ability to explore the physical and chemical properties of materials at the atomic scale. Molecular dynamics simulation is based on classical mechanics. The trajectory of each atom is calculated by solving the Newton motion equation, and the interaction between atoms is described by the empirical force field model. This technology can accurately capture and monitor the microscopic dynamic information of intermolecular interactions in the material system in real time and obtain its mechanism of action and evolution law. It has been widely used in the research of materials science and has become an indispensable cross-scale simulation tool for correlation analysis from microstructure to macroscopic properties. In the specific research practice, Mesfin Tsige et al. [38] carried out molecular dynamics simulation and theoretical calculations of a polymer reference interaction site model for C_48_F_98_ oligomer of polytetrafluoroethylene PTFE at 500 and 600 K. The consistency of theory and simulation is equivalent to the results of early research on polyolefin melt. The application of MD simulation on PTFE and its composites is more focused on the study of friction performance. Many scholars have studied the friction process and tribological performance mechanism of SiO_2_ [39], graphene (Gr) [40], Fe [41] and polytetrafluoroethylene (PTFE) composites using molecular dynamics (MD) simulation. Zhen Dong et al. [42] used molecular dynamics (MD) simulation to determine the optimal sintering temperature of carboxyl-functionalized graphene (GNS-COOH)-modified polyetheretherketone (PEEK)/polytetrafluoroethylene (PTFE) composites. The GNS-COOH/PEEK/PTFE composite model was constructed, the effects of different sintering temperatures on its mechanical and tribological properties were simulated, and its atomic mechanism was analyzed. Although MD simulation technology has made remarkable progress in the study of friction properties of PTFE and its composites, the research on its mechanical properties is still relatively scarce.

In this work, three kinds of PTFE-Cu composite samples with different densities are prepared. The crystallinity parameters of the PTFE matrix in each sample are characterized by experiments. The quasi-static compression test is carried out to quantitatively explore the macroscopic mechanical response characteristics of PTFE-Cu composites, and the influence of crystallinity on mechanical properties is analyzed. Finally, based on the molecular dynamic simulation method, we discuss the microstructure evolution of PTFE-Cu composites under compression load and reveal the molecular dynamics mechanism of deformation failure, which provide a multi-scale study for understanding the structure–activity relationship of PTFE-Cu composites.

## 2. Materials

In this study, DuPont 7A PTFE (30 μm, Guangdong Dongguan Zhonglian Plasticizing Co., Dongguan, China) and oxygen-free high-conductivity copper (OFHC, 3~5 μm, Hebei Xingtai Zhongye Xindun alloy Co., Xingtai, China) were selected. The mechanical properties test samples of PTFE-Cu materials were prepared by hot-pressing sintering process. Firstly, PTFE powder and copper powder were uniformly mixed by shear mixer. The mixed powder was dried and put into the mold. The pressure was applied while sintering in the high-temperature sintering furnace. The whole preparation process was carried out in argon atmosphere, and the applied pressure was constant pressure.

For composite materials composed of more than two materials, the actual density *ρ* of the material sample can be obtained by using the buoyancy method of Archimedes principle and MH-300G multifunctional density balance [34]. The mass fraction, volume and density of PTFE are recorded as $C_{PTFE}$, $V_{PTFE}$ and $\rho_{PTFE}$, respectively. The mass fraction, volume and density of Cu are recorded as $C_{Cu}$, $V_{Cu}$ and $\rho_{Cu}$, respectively. The total mass and final density of the sample are recorded as *m* and *ρ*, respectively. The volume of PTFE and Cu in the PTFE-Cu composite sample is expressed as:

(1) $$V_{PTFE}=m\cdot C_{PTFE}/\rho_{PTFE}$$

(2) $$V_{Cu}=m\cdot C_{Cu}/\rho_{Cu}$$

The maximum theoretical density of the sample can be calculated using the following formula:

(3) $$\rho =m/\left(V_{PTFE}+V_{Cu}\right)=\rho_{PTFE}\cdot \rho_{Cu}/\left(C_{PTFE}\cdot \rho_{Cu}+C_{Cu}\cdot \rho_{PTFE}\right)$$

In order to explore the influence of different ratios of PTFE and Cu on the properties of composite materials, three different density ratios were designed in this study. The density and mass ratio of each material are listed in Table 1.

## 3. Methods

### 3.1. Experiment of Differential Scanning Calorimetry

Differential scanning calorimetry (DSC) is used to control the sample at a certain temperature program, observe the change process of the heat flow power difference between the sample and the reference substance with temperature or time, so as to obtain the heat absorption, heat release, specific heat change and other related thermal effect information of the sample in the process of temperature program, and calculate the heat absorption and heat release and characteristic temperature of the thermal effect.

In this study, the experimental instrument for testing the thermodynamic properties of PTFE-Cu composites is Mettler DSC3 differential scanning calorimeter produced by METTLER TOLEDO Group in Switzerland. It can measure the specific heat, melting temperature, heat absorption and release, phase transition temperature, crystallization temperature, glass transition temperature, crystallinity and other thermodynamic parameters of the material. The test conditions were as follows: 20 mg of PTFE-Cu composite sample was placed in a crucible, and the crucible was heated at a heating rate of 40 °C/min in an air atmosphere. After reaching 380 °C, it was kept for 5 min, and then slowly cooled to 10 °C at a rate of 1 °C/min. Then, the sample was heated to 380 °C at a rate of 10 °C/min for five minutes, and finally cooled at a rate of 10 °C/min to observe the heat flow of the sample with temperature change. In this experiment, the sample was rapidly heated and then slowly cooled, so that the molecular chains inside the sample melt had enough time to crystallize regularly, avoiding cold crystallization during the second heating process, thus affecting the experimental results.

### 3.2. Quasi-Static Compression Test Conditions

In this study, the quasi-static compression of PTFE-Cu composites at room temperature was carried out by using CMT5105 microcomputer-controlled electronic universal testing machine produced by MTS Systems (China) Company in Hangzhou, China. The universal testing machine can carry out various basic mechanical property tests of materials at room temperature, and the maximum load of force loading can reach 100 kN.

Quasi-static compression tests were carried out on PTFE-Cu composite specimens with different densities. According to the selected strain rate and the height of the compression specimen, the compression rate of the universal testing machine was determined. The calculation formula is as follows:

(4) $$\dot{\varepsilon }=v/H$$

**Figure 1:**
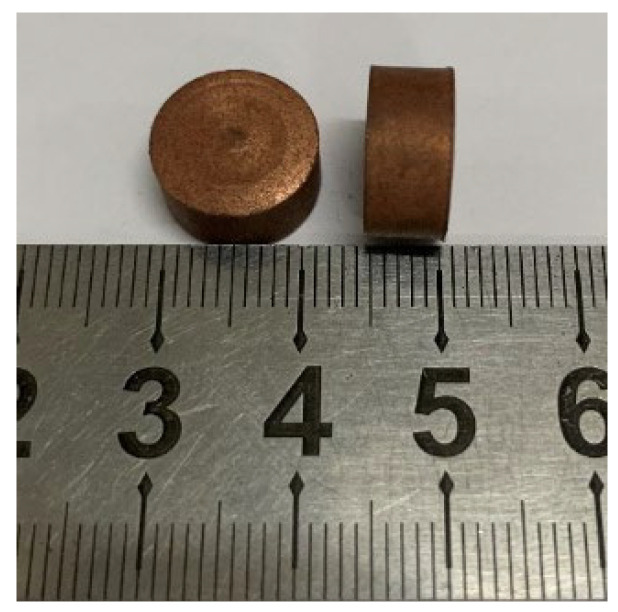
Physical figure of quasi-static compression specimen.

In the formula, $\dot{\varepsilon }$ is the loading strain rate of the sample, *v* is the compression speed of the universal testing machine, and *H* is the initial height of the sample. In this test, the diameter of the sample is 12 mm, and the initial height is 6 mm, as shown in Figure 1. According to Reference [43], the optimum strain rate of the quasi-static compression test of PTFE-Cu samples prepared by hot pressing sintering process is 0.01 s^−1^, so the quasi-static compression strain rate of the three samples in this study is set to 0.01 s^−1^. Therefore, the compression rate of the universal testing machine during the test is 3.6 mm/min. The test data are recorded by a force sensor and a displacement sensor, and the engineering stress and engineering strain of the specimen are calculated using the following formula:

(5) $$\sigma_{E}=F/\left[\pi {\left(D/2\right)}^{2}\right]=4F/\left(\pi D^{2}\right)$$

(6) $$\varepsilon_{E}=\left(H-h\right)/H$$

where *F* is the compression load, *D* is the original diameter of the sample, *H* is the original height of the sample, and *h* is the instantaneous height of the sample at a certain moment under the compression load. Then, the formula for calculating the true stress and true strain of the specimen under quasi-static pressure is:

(7) $$\sigma_{T}=Fh/\left[\pi {\left(D/2\right)}^{2}H\right]=\left(\pi D^{2}\sigma_{E}h/4\right)/\left[\pi {\left(D/2\right)}^{2}H\right]=\sigma_{E}h/H=\sigma_{E}(1-\varepsilon_{E})$$

(8) $$\varepsilon_{T}=\int_{l}^{l_{0}}d\varepsilon =\int_{l}^{l_{0}}\left(dl/l\right)=\ln\left(l_{0}/l\right)=\ln1/\left(1-\varepsilon_{E}\right)=-\ln\left(1-\varepsilon_{E}\right)$$

### 3.3. Molecular Dynamics Simulation

Condensed-phase Optimized Molecular Potentials for Atomistic Simulation Studies (COMPASS) force field is a condensed-state optimized molecular force field for atomic simulation research. It is the first ab initio force field to accurately predict the performance of polymers [44]. The force field systematically integrates the multi-body coupling mechanism of bond interaction, non-bond interaction and polarization effect through a quantum chemical parameterization method, and it constructs an all-atom potential function that can accurately describe the potential energy surface of complex systems. It shows excellent quantitative prediction ability in predicting the thermodynamic properties, mechanical properties and transmission characteristics of polymer materials, and its prediction accuracy is highly consistent with the experimental observations. In the COMPASS force field, the total potential energy consists of a bond interaction term and a non-bond interaction term. The bond interaction has bond stretching potential energy, bond angle bending potential energy, dihedral angle torsion potential energy, and off-plane vibration potential energy. Non-bond interactions include van der Waals force, hydrogen bond energy and electrostatic interaction (Coulomb function) [45]. The form of non-bond interaction potential energy is Lennard–Jones (LJ) potential energy, and its expression is as follows:

(9) $$U\left(r\right)=4\varepsilon \left[{\left(\sigma /r\right)}^{9}-{\left(\sigma /r\right)}^{6}\right]$$

Here, *ε* denotes the depth of the potential well, that is, the difference between the lowest point of the potential energy and the potential energy of 0; *σ* denotes the equilibrium distance between two atoms; *r* denotes the distance between two atoms in any state. This force field has been proved to be able to describe the mechanical properties of polymers [46].

**Figure 2:**
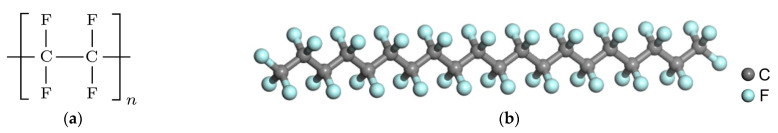
PTFE model: (**a**) molecular structure; (**b**) molecular chain model.

PTFE-Cu composite material model was constructed in Materials Studio 23.1 (MS) software developed by Accelrys, San Diego, US. PTFE is a chain polymer material composed of multiple repeating units, and its molecular structure is shown in Figure 2a. When the number of repeat units *n* = 10, the molecular chain model is shown in Figure 2b.

**Figure 3:**
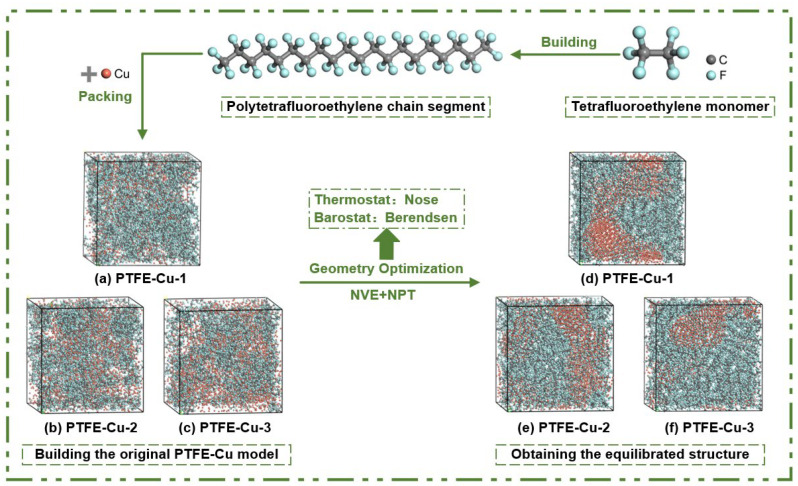
Modeling process of molecular structure model.

The molecular model size of the three materials is about 5 nm × 5 nm × 5 nm. The PTFE-Cu composite models were constructed by using the Amorphous Cell (AC) module of MS software. Based on the Monte Carlo rules, the PTFE chain and Cu were randomly filled into a periodic box with a size of 5 nm × 5 nm × 5 nm by using the packing method. The densities of PTFE-Cu composites were 3 g/cm^3^, 3.5 g/cm^3^ and 4 g/cm^3^, respectively. The initial temperature of modeling is 300 K. The molecular structure model construction process of the three materials is shown in Figure 3.

Firstly, the smart method was used to minimize the energy of the three models in 50,000 steps. The energy convergence threshold was set to 10^−5^ kcal/mol, and the force convergence threshold was set to 0.001 kcal/mol/Å. Then, five cycles of annealing simulation were carried out on the model, the temperature was 300~500 K, the micro canonical ensemble (NVE) was used, the temperature gradient was 50 K, and the total time was 1 ns. Finally, the simulation calculation of 1 ns was carried out under the isothermal isobaric ensemble (NPT). In the optimization process, Nose is selected as the temperature control mode, and Berendsen is selected as the pressure control mode. Nose–Hoover thermostat and Berendsen barostat algorithm are applied in the temperature and pressure conversions.

The software used in the crystallization and compression simulation is the Large-scale Atomic/Molecular Massively Parallel Simulator (LAMMPS) developed by Sandia National Laboratory. The software version used in this study is LAMMPS-64bit-29Aug2024-MSPI. The model of the last frame of NPT dynamics was imported into LAMMPS software. For crystallization simulation experiment, the initial MD simulation model was equilibrated for 50 ps at 900 K, to make sure that the PTFE matrix reached the liquid state. Then, the temperature of the system was decreased from 900 K to 300 K at a cooling rate 0.1 K/ps to simulate the crystallization process. The simulations were carried out under the isothermal–isobaric (NPT) ensemble with a time step of 1 fs. Finally, when the temperature dropped to 300 K, the simulation system was relaxed for 50 ps so that it could be in the equilibrium state. The uniaxial compression method was used for compression simulation calculation. The specific process involved minimizing the energy of the model first, so that the energy of the system reached a relatively stable state. Then, the uniaxial compression simulation of the model was carried out under the NPT ensemble. The compression direction was Z direction, and the pressure in two directions (X, Y direction) perpendicular to Z direction was set to 1 atm. The simulation process was realized by the fix deform command, and the tensile strain rate was set to 1 × 10^−4^ ps^−1^. In the whole simulation process, the calculation methods of electrostatic interaction and van der Waals force were Ewald and Atom-Based, respectively. The temperature was 300 K, the pressure was 1 atm, the cut-off radius was set to 1.25 nm, the time step was 1 fs, and the boundary condition was periodic.

## 4. Results and Discussion

### 4.1. Analysis of DSC Test Results

**Figure 4:**
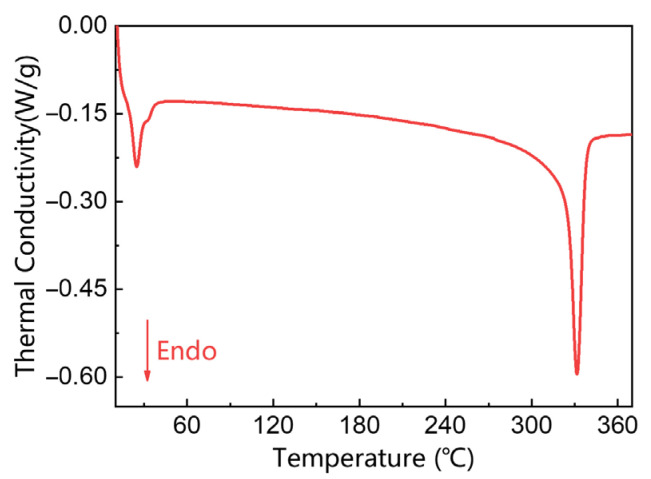
DSC curve of PTFE-Cu-1.

**Figure 5:**
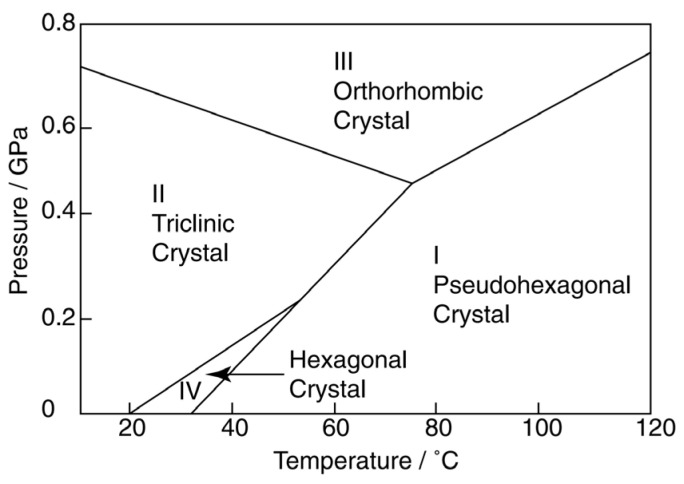
Phase diagram of PTFE at low pressure [16].

The DSC curve of the PTFE-Cu-1 sample at the second heating is shown in Figure 4. Endo represents the endothermic process. Combined with the analysis of the relevant literature [16], the first downward peak in Figure 4 is the solid–solid phase transition process of PTFE in the composite material. The helically arranged triclinic crystal system in PTFE is transformed into a hexagonal crystal system, which is a reversible two-way transition, corresponding to the transition process of PTFE from phase Ⅱ to phase Ⅳ in Figure 5 (19 °C). Reading the data in Figure 4, the starting point of the phase transition is 17.32 °C, which is close to the transition temperature of the Ⅱ → Ⅳ phase. The second peak represents the melting process of the polymer, and the peak temperature is 327.6 °C.

The solid–solid phase transition starting temperature data of the three materials are listed in Table 2. The analysis shows that the phase transition process occurs near the characteristic phase transition temperature (about 19 °C) of PTFE in the three sample systems. The phase transition onset temperature of the PTFE-Cu-1 sample moves forward (lower than 19 °C), while the phase transition onset temperature of PTFE-Cu-2 and PTFE-Cu-3 samples is higher than 19 °C. This phenomenon indicates that the addition of Cu powder will affect the phase transition temperature of PTFE. Within a certain range of addition content, Cu powder can induce a decrease in phase transition temperature; however, when the content of copper powder exceeds a certain value, it may cause the hysteresis effect of the phase transition temperature.

**Figure 6:**
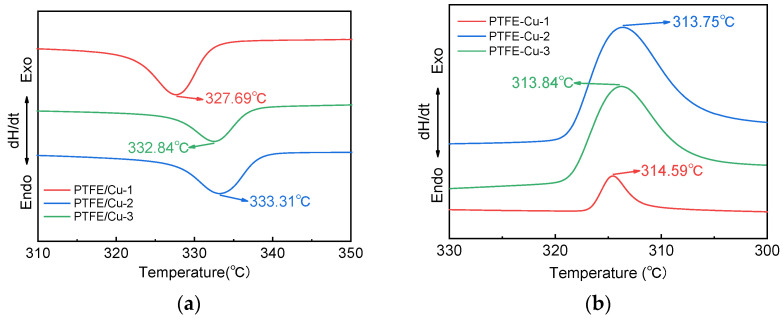
DSC curves of three samples: (**a**) melting stage; (**b**) crystallization stage.

**Figure 7:**
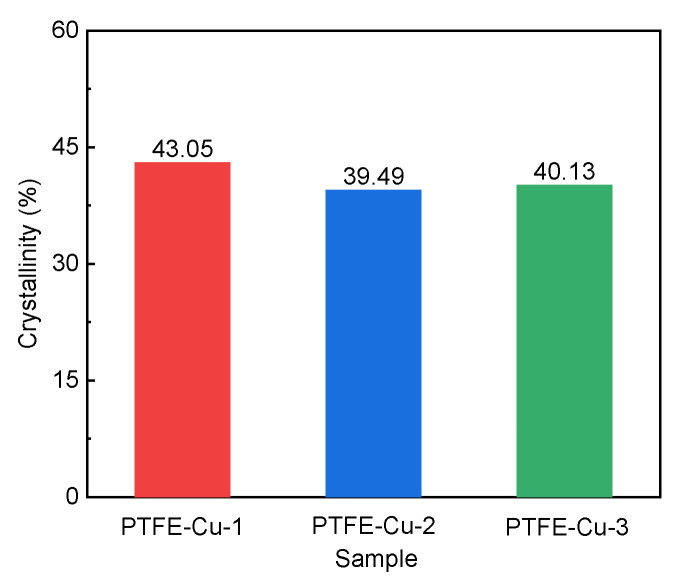
Crystallinity of three samples.

DSC curves of the three materials in the melting stage and the crystallization stage are shown in Figure 6a,b, respectively (the melting stage described in this section only indicates the melting of PTFE, and Cu does not melt at this temperature). The first sample to complete the melting process was PTFE-Cu-1, which reached the melting peak at 327.69 °C, followed by PTFE-Cu-3 and PTFE-Cu-2. According to the melting enthalpy, the crystallinity *X_C_* of PTFE in the three samples can be calculated. The calculation formula is as follows:

(10) $$X_{C}={∆H}_{f}/{∆H}_{f0}$$

where ${∆H}_{f}$ is the melting enthalpy of the sample, J/g; ${∆H}_{f0}$ is the theoretical melting enthalpy of completely crystallized matter, J/g. The theoretical melting enthalpy of PTFE in this study is 82 J/g. The crystallinity of the three samples calculated via Equation (10) was 43.05%, 39.49% and 40.13%, respectively, as shown in Figure 7, indicating that the addition of copper powder made the crystallinity of PTFE decrease first and then increase.

Based on the crystallization heat, the relationship between the number-average molecular weight and the crystallization enthalpy can be established:

(11) $$M_{n}={∆H}_{c}^{-5.16}\times 2.1\times 10^{10}$$

In the formula, ${∆H}_{c}$ is the crystallization enthalpy, J/g. The number-average molecular weight calculation results of the three samples are shown in Table 3.

The crystallization enthalpy and the number-average molecular weight show a significant negative correlation; that is, with a decrease in molecular weight, the conformational flexibility of the molecular chain is enhanced, the entanglement effect between the intramolecular and intermolecular is weakened, and the activity of the molecular chain is enhanced, which provides a more favorable condition for the crystal transformation process, and the crystal transformation occurs more easily. Based on this mechanism, the PTFE-Cu-1 composite exhibits the earliest crystallization initiation behavior during the cooling crystallization process, and its crystallization peak temperature is 314.5 °C. Subsequently, PTFE-Cu-3 and PTFE-Cu-2 composites crystallized in turn. Although the crystallization peak temperatures of the three composites were all concentrated near 314 °C, there was a slight deviation. This phenomenon may be related to the content of Cu powder in the system.

Due to the regular conformation, excellent symmetry and good flexibility of the molecular chain, the macromolecular chain segments of the pure PTFE system can efficiently migrate to the surface of the crystal nucleus and form an orderly arrangement during the crystallization process. At the same time, the entanglement between the molecular chains is weak, which further promotes the crystallization process. The addition of Cu powder causes local damage to the crystal structure of PTFE, hindering the growth of complete crystals, which can only form fine grains dispersed in the amorphous structure, resulting in the original complete crystalline segment in PTFE becoming a random conformation. Therefore, the crystallinity of PTFE-Cu composites is significantly lower than that of pure PTFE materials.

With an increase in the density of PTFE-Cu composites, the content of Cu powder increases, and the characteristic peak temperature that meets the crystallization conditions decreases. The ratio of PTFE and Cu in the sample with a density of 3.5 g/cm^3^ can be approximately regarded as 1:1. When the ratio exceeds this ratio, the content of Cu powder exceeds that of PTFE, and the crystallization peak temperature increases. This is because when the ratio of the content of the two is greater than 1, the PTFE phase in the composite system gradually transforms into a dispersed phase wrapped by the Cu matrix, rather than the traditional continuous matrix phase. This phase inversion leads to an enhancement in the constraint of the PTFE chain segment. The degree of chain disorder is relatively reduced, but it promotes increased crystallinity again, accompanied by a corresponding increase in the crystallization peak temperature. In addition, the thermodynamic analysis of the crystallization process shows that the increase in the crystallization temperature can significantly enhance the thermal motion ability of the polymer chain segment and provide more favorable kinetic conditions for the formation of crystal nucleus and grain growth, thus promoting an increase in crystallinity. This understanding is consistent with the aforementioned change trend of the crystallization peak temperature, indicating that the composition ratio of PTFE-Cu composites can regulate the crystallization behavior.

### 4.2. Quasi-Static Compression Test Results

#### 4.2.1. Quasi-Static Compressive Mechanical Properties

**Figure 8:**
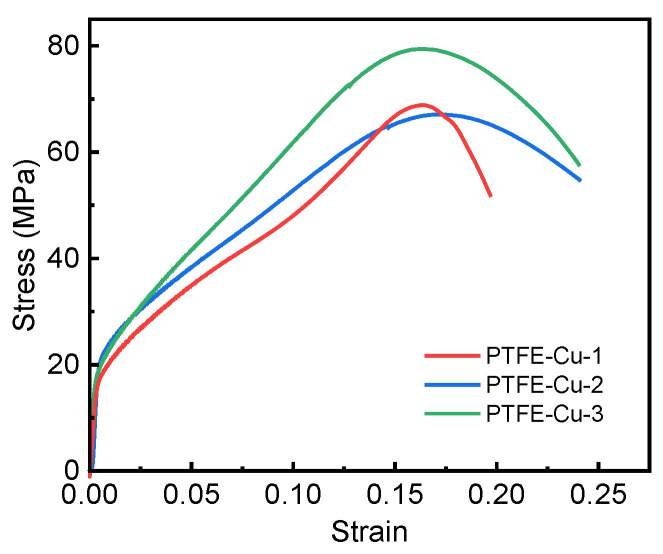
The stress–strain curves of the three specimens at a strain rate of 0.01 s^−1^.

In this study, three groups of effective tests are carried out for each sample and the average value is obtained. The stress–strain curve is shown in Figure 8. It can be seen from the curve that the material has gone through three stages: elastic deformation stage, plastic strengthening stage and failure stage. In the initial stage of loading, there is an approximate linear relationship between the stress and strain of the three samples; that is, the elastic deformation of the material occurs. This stage is shorter, and the material can only produce small deformation under the action of large stress. If the load is unloaded at this stage, the sample can almost return to the original length. When the stress reaches the yield point of the material, the specimen begins to undergo plastic deformation. As the stress increases, the strain increases rapidly, and the material is continuously strengthened during the plastic deformation process until the deformation reaches the ultimate strain of the material. Cracks are generated inside the specimen. At this time, the stress decreases as the strain continues to increase, and the crack propagates unstably until the material completely fails.

**Figure 9:**
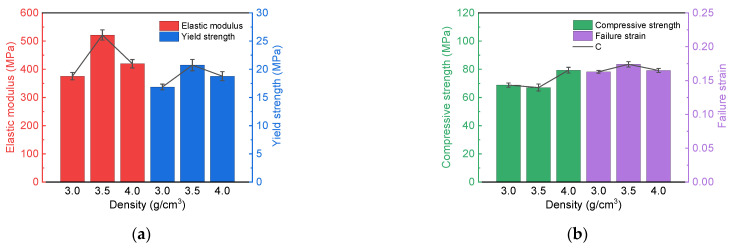
Quasi-static mechanical properties of PTFE-Cu samples: (**a**) elastic modulus and yield strength parameters; (**b**) compressive strength and failure strain parameters.

According to the quasi-static test, the elastic modulus, yield strength, compressive strength and failure strain of PTFE-Cu materials under three densities can be obtained. Table 4 shows the mechanical properties of three PTFE-Cu samples obtained from the quasi-static compression test. Each parameter is taken from the average value of the three repeated tests, and the standard errors of the corresponding parameters are calculated, as shown in Figure 9.

The elastic modulus of the material reflects its ability to resist elastic deformation in the elastic range. Obviously, the elastic modulus of the PTFE-Cu-2 sample is the largest, which is much higher than that of the other two materials, reaching 521.27 MPa. It can be seen from the thermodynamic performance analysis that when the density is 3.5 g/cm^3^, the crystallinity of PTFE in the material is the smallest, and the proportion of the amorphous phase is higher. The material mainly undergoes approximate elastic deformation before reaching the yield point, and the elastic deformation is mainly controlled by the amorphous region of the crystalline polymer. Therefore, the lower the crystallinity of the PTFE-Cu material, the higher the elastic modulus.

It can be seen from Figure 9a that with the gradual increase in material density, the yield strength shows a trend of increasing first and then decreasing. This phenomenon can be explained by the theory of material crystallography: low-crystallinity materials show more significant lattice distortion ability in the elastic deformation stage, and the lattice dislocation motion of low-crystallinity materials is constrained by the amorphous phase, thus significantly improving the yield strength. The higher crystallinity leads to an increase in lattice defect density and the formation of a local stress concentration zone, which accelerates the generation and development of plastic deformation.

Figure 9b shows the histogram distribution of compressive strength and failure strain of each PTFE-Cu sample. When the density of the sample reaches 4 g/cm^3^, the compressive strength has the maximum, and the specific value is 79.40 MPa. This phenomenon can be attributed to the increase in the density of the material, which leads to an increase in the content of the larger metal Cu particles in the interior. Under quasi-static compressive loading, the skeleton support of these Cu particles becomes more and more significant, thereby significantly enhancing the compressive strength of the material [47].

#### 4.2.2. Failure Mode Analysis

**Figure 10:**
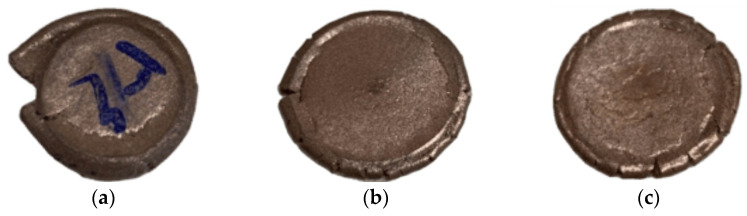
Typical failure morphology of compression specimen: (**a**) PTFE-Cu-1; (**b**) PTFE-Cu-2; (**c**) PTFE-Cu-3.

The main failure modes of polymer and metal composite specimens are axial splitting failure and shear failure [48]. It can be seen from Figure 10 that the typical failure mode of the PTFE-Cu compression specimen is axial splitting failure. There is a significant stress concentration phenomenon between the PTFE matrix and the metal particle interface. When the stress concentration exceeds the ultimate strength of the material, the crack will initiate and expand rapidly in the matrix. The propagation path of the crack eventually tends to be approximately parallel to the direction of the compressive load. From the macroscopic level, the side of the specimen shows different degrees of crack morphology and even forms a split failure with both ends connected.

In the PTFE-Cu-1 composite sample, the content of PTFE is as high as 63%, which becomes the key component that dominates the mechanical response under compressive load. As the load continues to be applied, the bearing capacity of the material decays rapidly, and the crack growth rate accelerates significantly. During the compression process, the contact interface between the indenter and the sample induces end effect due to friction, resulting in relatively small deformation at both ends of the sample. At the same time, the outer surface of the middle part of the sample shows a drum-shaped expansion characteristic, which, in turn, causes a radial tensile effect. Finally, the material exhibits a typical shear tearing morphology, as shown in Figure 10a. In the PTFE-Cu-2 and PTFE-Cu-3 samples, the content of metal particles increases, which leads to an increase in stress concentration between the matrix and the particle interface, and the number of microcracks shows a significant growth trend. The probability of the end effect is reduced, leading to the disappearance of the radial stretching effect. Therefore, as shown in Figure 10b,c, the failure mode of the specimen is the axial splitting failure with both ends connected, and no shear tearing failure occurs.

### 4.3. Molecular Dynamics Simulation Results

#### 4.3.1. Crystallization Simulation Results

**Figure 11:**
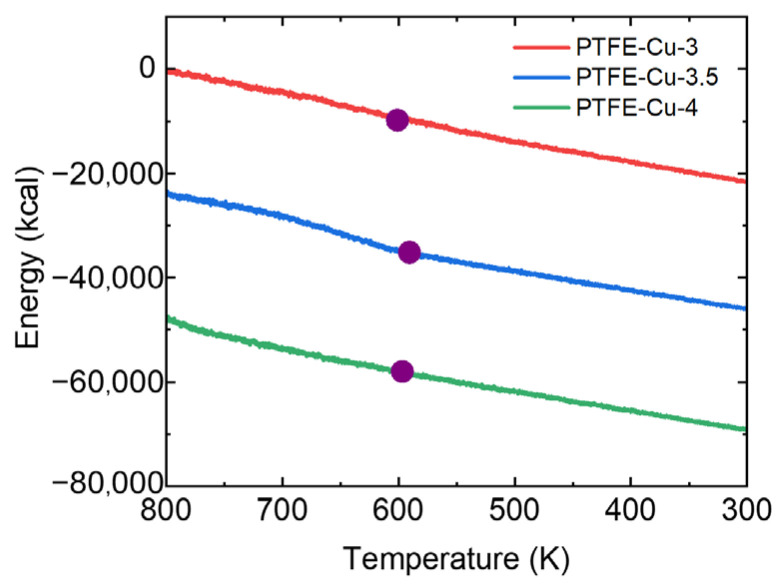
Temperature-dependent potential energy of PTFE-Cu during crystallization (the purple circle represents the deflection point of the curve).

Figure 11 shows the variation in the potential energy of the PTFE-Cu composite with temperature during crystallization. The potential energy monotonically decreases as the temperature decreases.

Deflections appear in the decline curve range from 600 K to 550 K, corresponding to the crystallization of PTFE. The errors in the crystallization peak temperature between the DSC experiment and crystallization simulation are listed in Table 5. The errors of the crystallization peak temperature of the DSC experiment and the crystallization simulation of the three materials are slight, which are 2.24%, 0.75%, 1.69%. In addition, the change trend of the crystallization peak temperature in the crystallization simulation is also consistent with the DSC experiment, indicating the effectiveness of the molecular dynamic simulation.

As a key physical quantity reflecting the microstructure characteristics of materials, the radial distribution function g(r) represents the ratio of the probability density of another molecule to the probability density of random distribution at a specific distance r around a molecule. By systematically analyzing the radial distribution function g(r) of the equilibrium conformational trajectory generated by molecular dynamics simulation, the interaction mechanism between PTFE and Cu interface system can be quantitatively analyzed, and its microscopic action mode and essential characteristics can be revealed.

**Figure 12:**
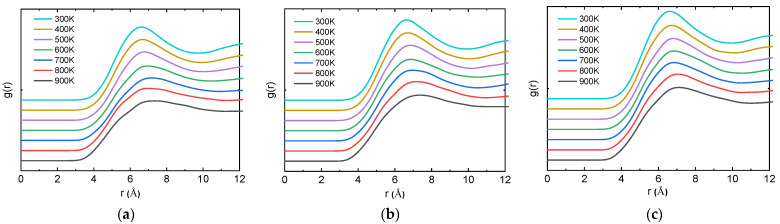
RDF curves of PTFE during crystallization. (**a**) PTFE-Cu-1; (**b**) PTFE-Cu-2; (**c**) PTFE-Cu-3.

The structural evolution of PTFE-Cu materials during the crystallization can be revealed by RDFs at different temperatures, as shown in Figure 12. As the temperature decreases, the peaks in RDFs become more obvious. The higher and sharper peaks indicate that the PTFE becomes more ordered at a lower temperature [49]. In addition, in Figure 12a–c, the peaks of the curves at 500 K and 600 K have changed significantly. Obviously, the curve peak at 500 K becomes steeper than the curve peak at 600 K, indicating that the crystallization temperatures of the three materials are between 500 K and 600 K. This is consistent with the previous results, indicating the reliability of the simulation.

#### 4.3.2. Compression Simulation Results

**Figure 13:**
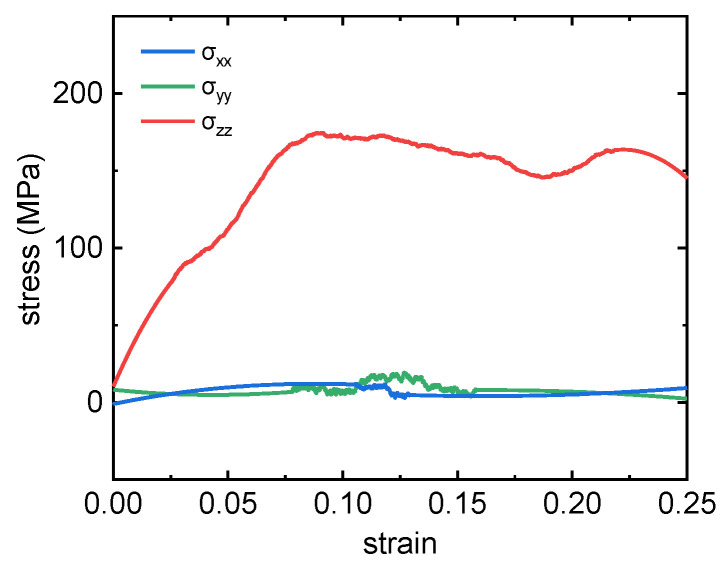
Compression stress–strain curve of PTFE-Cu-1.

In order to verify the rationality of the simulation method, taking PTFE-Cu-1 as an example, the stress–strain curve during compression was calculated, as shown in Figure 13.

During the whole compression deformation process, the stress components *σ*_xx_ and *σ*_yy_ of PTFE-Cu-1 composites in the X-axis and Y-axis directions basically maintained a near-zero value, while the stress component *σ*_zz_ in the Z-axis direction changed with an increase in strain. The compressive strength of PTFE-Cu-1 is about 174 MPa, which is significantly higher than the test results in previous studies. This phenomenon can be attributed to the fact that in order to effectively save computing resources, a strain rate of up to 1 × 10^8^ s^−1^ is used in the simulation calculation process, which is far beyond the level set in the experiment. In view of the fact that the compressive strength of PTFE-Cu composites increases with an increase in the loading strain rate [16,23], the simulation results obtained in this study under higher strain rate conditions are within a reasonable expected range.

**Figure 14:**
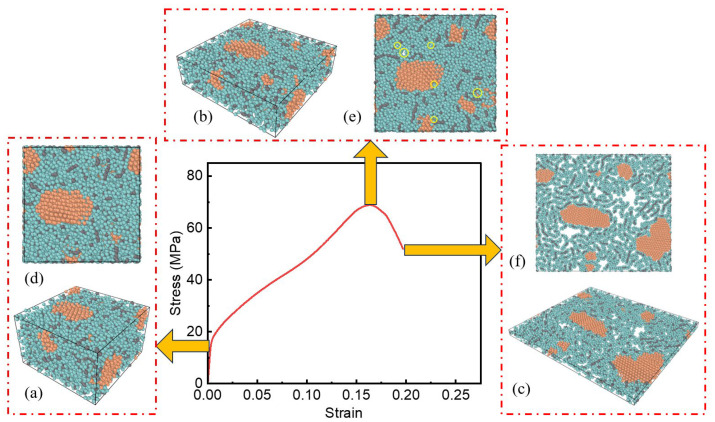
Internal microstructure of PTFE-Cu-1 sample: (**a**) material microstructure at the end of elastic stage; (**b**) material microstructure at the end of plastic strengthening stage; (**c**) material microstructure at the end of failure stage; (**d**) top view of material microstructure at the end of elastic stage; (**e**) top view of material microstructure at the end of plastic strengthening stage; (**f**) top view of material microstructure at the end of failure stage.

**Figure 15:**
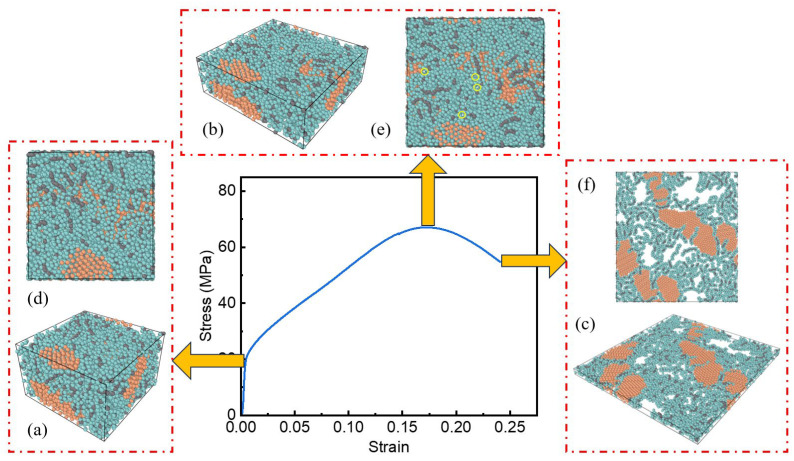
Internal microstructure of PTFE-Cu-2 sample: (**a**) material microstructure at the end of elastic stage; (**b**) material microstructure at the end of plastic strengthening stage; (**c**) material microstructure at the end of failure stage; (**d**) top view of material microstructure at the end of elastic stage; (**e**) top view of material microstructure at the end of plastic strengthening stage; (**f**) top view of material microstructure at the end of failure stage.

**Figure 16:**
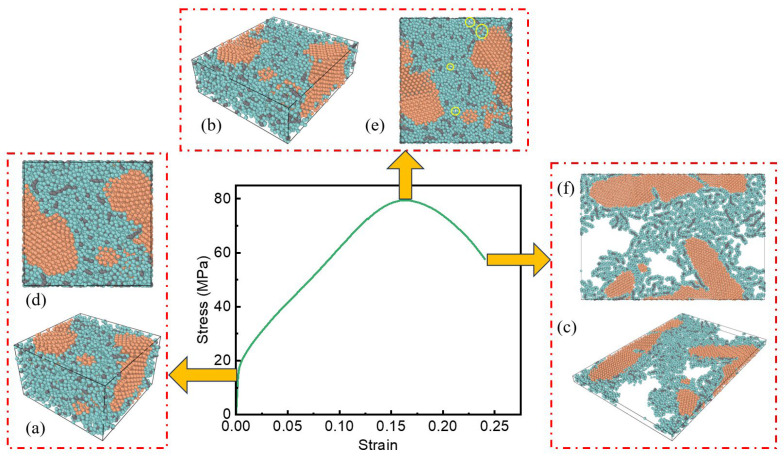
Internal microstructure of PTFE-Cu-3 sample: (**a**) material microstructure at the end of elastic stage; (**b**) material microstructure at the end of plastic strengthening stage; (**c**) material microstructure at the end of failure stage; (**d**) top view of material microstructure at the end of elastic stage; (**e**) top view of material microstructure at the end of plastic strengthening stage; (**f**) top view of material microstructure at the end of failure stage.

Figure 14a–c, Figure 15a–c and Figure 16a–c show the internal microstructure evolution characteristics of the three specimens under elastic deformation, plastic strengthening and failure critical state, respectively. In order to deeply analyze the anisotropic characteristics of the material during the deformation process, a two-dimensional top view along the loading direction (Figure 14d–f, Figure 15d–f and Figure 16d–f) is further obtained, which intuitively shows the orientation-dependent evolution of the microstructure at different deformation stages.

For the PTFE-Cu-1 sample, the PTFE molecule acts as a matrix to wrap the aggregated Cu clusters. In the elastic deformation stage, the particle spacing decreases, and the PTFE molecular chain undergoes elastic distortion. The chain segment movement leads to the shortening of the C-F bond length inside the molecule along the loading direction, the reversible deflection of the dihedral angle of the chain segment, and the transformation of the all-trans conformation to the spiral conformation. These changes will recover after removing the applied load. The Cu clusters and PTFE matrix form a synergistic deformation mechanism through interfacial coordination, and the chain segment movement of the PTFE molecular chain causes lattice distortion on the surface of the Cu clusters. The two coupled elastic distortion mechanisms lead to the anisotropic Poisson effect of the composite material at the macro scale.

In the plastic strengthening stage of the material, the dislocation-mediated plastic deformation mechanism dominates the microstructure evolution. The stress concentration caused by the applied load promotes the conformational rearrangement of the PTFE macromolecular segments, the van der Waals force balance between the chains is broken, and the entanglement network achieves local reconstruction through segment slip. At the same time, the atoms in the Cu cluster cross the lattice barrier through dislocation climbing and sliding mechanisms, resulting in the accumulation of lattice distortion energy and triggering grain boundary migration. In the process of material deformation, the orientation behavior of the polymer chain and the micro-motion of the metal lattice show a dynamic coupling effect, and the two form a unique deformation mechanism through synergy. This mechanism promotes the irreversible structural reorganization of materials at the molecular or atomic scale, which, in turn, manifests as permanent changes in shape or size at the macro scale.

With an increase in the strain level, the internal microstructure of the system changed significantly, and the pore phenomenon gradually appeared. Specifically, the pores are mainly formed at the interface between the PTFE molecular chain and the Cu atom cluster. At this microscopic phase boundary, due to the interface mismatch effect caused by the difference in the mechanical response of the two-phase material, local stress concentration leads to structural instability and the formation of initial pores. At the same time, due to the orientation effect inside the PTFE molecular chain, the entanglement network structure undergoes a disentanglement process, resulting in a decrease in the local density between the molecular chains, thereby generating pores. The continuous increase in the strain level leads to a significant increase in the interface mismatch effect and orientation effect. The accumulation of this effect eventually promotes the expansion and connectivity of the microscopic pores inside the material and then evolves into an obvious crack structure that can be observed on the macro scale.

By observing the diagrams in Figure 12, Figure 13 and Figure 14c,f, it can be found that the surface of the Cu atom cluster in the system shows the phenomenon of adsorbing a layer of the PTFE molecular chain. Due to the interface mismatch effect, the PTFE molecular chain in the outer layer is no longer limited to the adjacent area of the Cu atom cluster but has a significant orientation behavior, resulting in a more obvious gap structure between the Cu atom cluster and the PTFE molecular chain. As the density of the system increases, the relative content of Cu clusters in the system also increases. Correspondingly, the number of interfaces between PTFE molecular chains and Cu clusters shows an increasing trend. In the high-strain state, the interface mismatch effect will be further strengthened, and the resulting void formation phenomenon will be more serious.

On the other hand, the addition of Cu destroyed the original stable conformation of the PTFE molecular chain, which hindered the orderly orientation movement of the PTFE molecular chain and increased its internal defects during compression. The increase in Cu content enhances this hindrance, which leads to the aggravation of PTFE internal defects.

**Figure 17:**
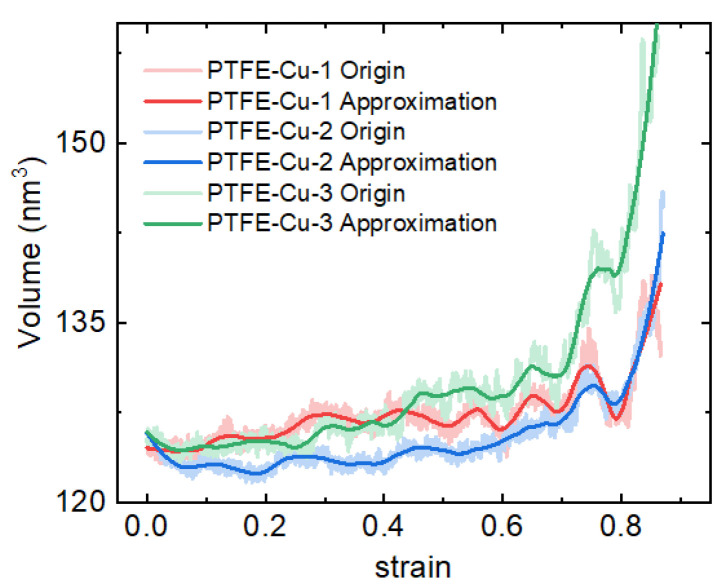
Volume change of the three materials in the simulation process.

Figure 17 shows the volume change of the three models during compression. It can be seen that in the elastic deformation stage, the volume of the three materials decreases slightly. It can be considered that the material is compacted and densified at this stage, resulting in a decrease in the volume of the model. In the subsequent plastic deformation stage, the material is continuously applied pressure. The interface between PTFE and Cu is weakly bonded, and the interface is peeled off during compression, forming microcracks or voids, resulting in an increase in volume and porosity. Finally, when the material breaks down, the volume increases sharply. The volumes of PTFE-Cu-1, PTFE-Cu-2 and PTFE-Cu-3 increase by 6.51%, 14.99% and 26.97%, respectively, compared with its initial volume. Correspondingly, it can be considered that when the three materials fail, the internal porosity is 6.51%, 14.99%, and 26.97%, respectively.

**Figure 18:**
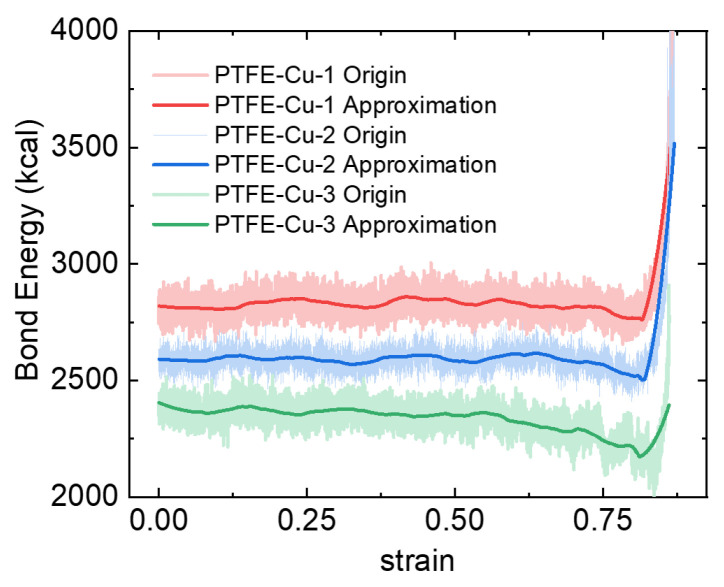
Bond energy change of the three materials in the simulation process.

The change trend of bond energy is shown in Figure 18. In the initial state, the bond energy of the three materials decreases with the decrease in PTFE content, which is due to the fact that the covalent bond energy in the polymer molecular chain is greater than the bond energy of the metal bond. The bond energy of the three materials is in a dynamic balance before the fracture failure, decreases before the failure, and then increases rapidly. The change in bond energy is usually related to the change in the distance between atoms. During the compression process, if the material undergoes plastic deformation, the arrangement of atoms may be disrupted, resulting in a decrease in bond strength. Dislocation movement leads to lattice distortion and disordered arrangement of atoms, resulting in a significant decrease in bond energy near the grain boundary. This behavior is more obvious in metal. Therefore, in the PTFE-Cu-3 material, due to the higher Cu content, the bond energy shows a slight downward trend during the plastic deformation process. Before the material is compressed to failure, the PTFE molecular chain is stretched and oriented along the stress direction. The stretching of the covalent bond leads to a rapid increase in bond energy. Therefore, the higher the PTFE content, the greater the increase in bond energy.

**Figure 19:**
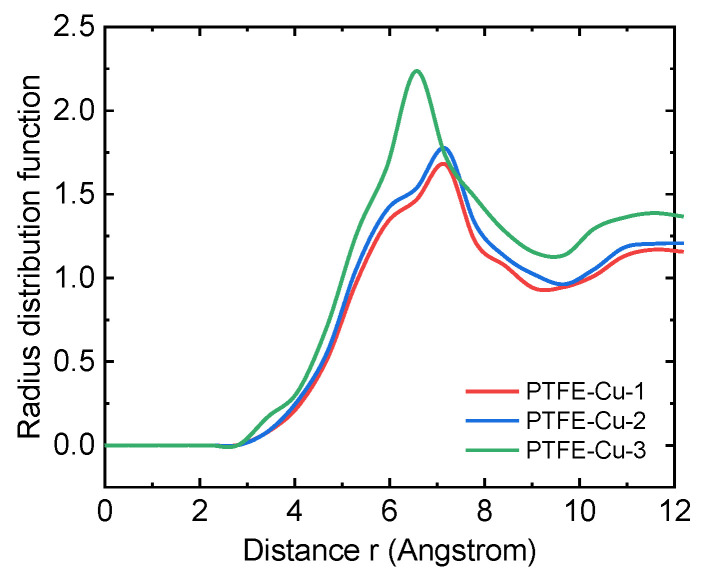
Radial distribution function of PTFE-Cu composites.

The atomic configuration of the PTFE molecular chain is composed of the main chain carbon (C) atom and the covalently bonded fluorine (F) side group atom. Its unique spiral conformation makes the main chain C atom completely coated by the F atom, forming a dense electron cloud barrier, which significantly weakens the orbital interaction probability with Cu. Based on this structural feature, this study focuses on the non-bond interaction mechanism between F atoms and Cu surfaces. The spatial correlation characteristics of the interaction between atoms are characterized by the functional relationship between the radial distribution function *g*(*r*) and the distance *r*, as shown in Figure 19. In the energy spectrum analysis of intermolecular interactions, the short-range peaks within 3.5 Å of the *g*(*r*) curve usually correspond to covalent bonds, coordination bonds or strong hydrogen bonding interactions, and the peaks in the 3.5~5 Å range mainly reflect the contribution of van der Waals forces [50]. However, the radial distribution function analysis in Figure 19 shows that the first coordination peak is located near 6.5 Å, which is significantly beyond the typical van der Waals range (<5 Å), indicating that the PTFE-Cu interface mainly forms long-range physical adsorption.

In order to further understand the interfacial properties of PTFE-Cu composites, the interfacial interaction energy between the PTFE matrix and Cu can be calculated. The calculation process is as follows [48]:

(12) $$E_{interaction}=E_{PTFE-Cu}-E_{PTFE}-E_{Cu}$$

where *E*_interaction_ represents the interaction energy between PTFE and Cu, *E*_PTFE-Cu_ represents the total potential energy of the composite, *E*_PTFE_ represents the potential energy of the PTFE matrix, and *E*_Cu_ represents the potential energy of Cu.

**Figure 20:**
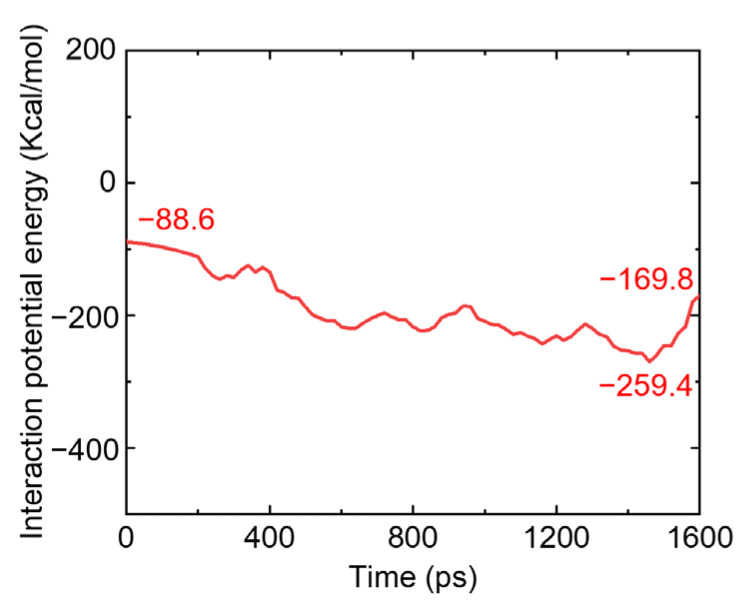
Interaction energy of PTFE-Cu composites.

Figure 20 shows the change in the interaction energy between the PTFE matrix and Cu during compression. According to the analysis, the interaction between the PTFE matrix and Cu shows an attractive characteristic, which is consistent with the microstructure characteristics of the PTFE molecular chain adsorbed on the surface of the Cu atomic cluster under compressive load. With an increase in the simulation time, the surface interaction energy shows a phased change: first, from −88.6 kcal/mol to −259.4 kcal/mol, then decreasing to −169.8 kcal/mol, meaning the interaction energy increased by 170.8 kcal/mol and then decreased by 89.6 kcal/mol. This is because, as the strain increases during the compression process, the length of the system in the Z direction gradually decreases, and the compactness of the arrangement between atoms gradually increases, so that the interaction distance between the PTFE matrix and Cu decreases and the interaction increases. With the continuous accumulation of strain, the adsorption effect of PTFE on Cu is weakened due to the orientation effect, which leads to a decrease in the interaction energy between PTFE and Cu, but it is still higher than the initial interaction energy. Therefore, a layer of the PTFE molecular chain is adsorbed on the surface of the Cu cluster in the failure state.

## 5. Conclusions

In this study, three kinds of PTFE-Cu composites with different densities (3.0 g/cm^3^, 3.5 g/cm^3^,4.0 g/cm^3^) were prepared. The crystallinity of the PTFE phase in each sample was quantitatively characterized by differential scanning calorimetry (DSC). The effect of crystallinity on the quasi-static compressive mechanical response of composites and the macroscopic failure mechanism of materials were further discussed. Combined with the molecular dynamics simulation method, the microstructure evolution rule and deformation failure mechanism of PTFE-Cu composites under compression load are obtained, which provides a theoretical basis for further understanding the force–structure coupling behavior of this kind of composite. The main conclusions are as follows:

(1) The composition ratio of PTFE-Cu composites affects the phase transformation and crystallization behavior of the material. The addition of Cu powder can affect the phase transition temperature of PTFE. Within a certain range, Cu powder can induce the phase transition temperature to decrease; however, when the content of copper powder exceeds the content of PTFE, the phase transition temperature may be delayed. The crystallinity of the three PTFE-Cu composites was 43.05%, 39.49% and 40.13%, respectively. With an increase in the copper powder content, the crystallinity decreased first and then increased, but it was lower than that of pure PTFE material.
(2) The quasi-static compression curve of PTFE-Cu composites has gone through three stages: elastic deformation stage, plastic strengthening stage and failure stage. The lower the crystallinity of the material, the higher the elastic modulus and yield strength, up to 521.27 MPa and 20.74 MPa, respectively. The side of the failure sample shows different degrees of crack morphology. The failure mode is mainly manifested as the axial splitting failure with two ends connected. The sample with a density of 3.0 g/cm^3^ also shows a shear tearing morphology.
(3) The coupling elastic distortion mechanism of the chain segment motion of the PTFE molecular chain and the lattice distortion of the Cu atom cluster leads to the anisotropic Poisson effect of the composite material at the macro scale. The synergistic deformation mechanism of the dynamic coupling of polymer chain orientation and metal lattice motion promotes the irreversible structural reorganization of the material at the molecular or atomic scale, which, in turn, manifests as permanent changes in shape or size at the macro scale. In the high-strain state, the interface mismatch effect is further strengthened, which leads to the formation of voids. The addition of Cu hinders the orderly orientation movement of its molecular chain, which leads to the internal defects of PTFE.
(4) When the three materials fail, the internal porosity is 6.51%, 14.99%, and 26.97%, respectively. The bond energy of PTFE-based composites initially decreases with lower PTFE content but rapidly increases before compressive failure as covalent bond stretching in PTFE molecular chains dominates, with higher PTFE content yielding greater energy enhancement. The radial distribution function shows that the first coordination peak is located near 6.5Å, which is significantly beyond the typical van der Waals range (<5 Å), indicating that the interaction of the PTFE-Cu interface is physical adsorption.

## Figures and Tables

**Table 1:** Ratio of PTFE-Cu samples.

No.	Theoretical Density (g/cm^3^)	Actual Density (g/cm^3^)	PTFE/wt%	Cu/wt%
PTFE-Cu-1	3.0	2.97	63	37
PTFE-Cu-2	3.5	3.54	49.5	50.5
PTFE-Cu-3	4.0	3.96	40	60

**Table 2:** The solid–solid phase transition starting temperature and melting peak temperature of the three samples.

Sample No.	PTFE/Cu-1	PTFE/Cu-2	PTFE/Cu-3
Phase transition onset/°C	17.32	19.50	19.20
Melting peak temperature/°C	327.69	333.31	332.84

**Table 3:** Number-average molecular weight calculation results and crystallization peak temperature of three samples.

Sample No.	PTFE/Cu-1	PTFE/Cu-2	PTFE/Cu-3
Number-average molecular weight	5.50 × 10^6^	7.28 × 10^7^	6.61 × 10^7^
Crystallization peak temperature/°C	314.59	313.75	313.84

**Table 4:** Quasi-static mechanical properties of PTFE-Cu samples.

Sample No.	Density (g/cm^3^)	Elastic Modulus (MPa)	Yield Strength (MPa)	Compressive Strength (MPa)	Failure Strain
PTFE-Cu-1	3	375.61	16.86	68.81	0.163
PTFE-Cu-2	3.5	521.57	20.74	67.05	0.174
PTFE-Cu-3	4	419.68	18.78	79.40	0.165

**Table 5:** Crystallization peak temperature between the DSC experiment and crystallization simulation.

Sample No.	PTFE/Cu-1	PTFE/Cu-2	PTFE/Cu-3
DSC experiment	587.74 K	586.90 K	586.99 K
Crystallization simulation	600.90 K	591.32 K	596.94 K
Error	2.24%	0.75%	1.69%

## Data Availability

The original contributions presented in this study are included in the article. Further inquiries can be directed to the corresponding author.
